# Activation of COX-2/PGE_2_ Promotes Sapovirus Replication via the Inhibition of Nitric Oxide Production

**DOI:** 10.1128/JVI.01656-16

**Published:** 2017-01-18

**Authors:** Mia Madel Alfajaro, Jong-Soon Choi, Deok-Song Kim, Ja-Young Seo, Ji-Yun Kim, Jun-Gyu Park, Mahmoud Soliman, Yeong-Bin Baek, Eun-Hyo Cho, Joseph Kwon, Hyung-Jun Kwon, Su-Jin Park, Woo Song Lee, Mun-Il Kang, Myra Hosmillo, Ian Goodfellow, Kyoung-Oh Cho

**Affiliations:** a Laboratory of Veterinary Pathology, College of Veterinary Medicine, Chonnam National University, Gwangju, Republic of Korea; b Division of Life Science, Korea Basic Science Institute, Seoul, Republic of Korea; c Bioindustry Research Center, Korea Research Institute of Bioscience and Biotechnology, Daejeon, Republic of Korea; d Division of Virology, Department of Pathology, University of Cambridge, Cambridge, United Kingdom; Instituto de Biotecnologia/UNAM

**Keywords:** caliciviruses, cyclooxygenases, nitric oxide, prostaglandin E_2_, sapovirus

## Abstract

Enteric caliciviruses in the genera Norovirus and Sapovirus are important pathogens that cause severe acute gastroenteritis in both humans and animals. Cyclooxygenases (COXs) and their final product, prostaglandin E_2_ (PGE_2_), are known to play important roles in the modulation of both the host response to infection and the replicative cycles of several viruses. However, the precise mechanism(s) by which the COX/PGE_2_ pathway regulates sapovirus replication remains largely unknown. In this study, infection with porcine sapovirus (PSaV) strain Cowden, the only cultivable virus within the genus Sapovirus, markedly increased COX-2 mRNA and protein levels at 24 and 36 h postinfection (hpi), with only a transient increase in COX-1 levels seen at 24 hpi. The treatment of cells with pharmacological inhibitors, such as nonsteroidal anti-inflammatory drugs or small interfering RNAs (siRNAs) against COX-1 and COX-2, significantly reduced PGE_2_ production, as well as PSaV replication. Expression of the viral proteins VPg and ProPol was associated with activation of the COX/PGE_2_ pathway. We observed that pharmacological inhibition of COX-2 dramatically increased NO production, causing a reduction in PSaV replication that could be restored by inhibition of nitric oxide synthase via the inhibitor *N*-nitro-l-methyl-arginine ester. This study identified a pivotal role for the COX/PGE_2_ pathway in the regulation of NO production during the sapovirus life cycle, providing new insights into the life cycle of this poorly characterized family of viruses. Our findings also reveal potential new targets for treatment of sapovirus infection.

**IMPORTANCE** Sapoviruses are among the major etiological agents of acute gastroenteritis in both humans and animals, but little is known about sapovirus host factor requirements. Here, using only cultivable porcine sapovirus (PSaV) strain Cowden, we demonstrate that PSaV induced the vitalization of the cyclooxygenase (COX) and prostaglandin E_2_ (PGE_2_) pathway. Targeting of COX-1/2 using nonsteroidal anti-inflammatory drugs (NSAIDs) such as the COX-1/2 inhibitor indomethacin and the COX-2-specific inhibitors NS-398 and celecoxib or siRNAs targeting COXs, inhibited PSaV replication. Expression of the viral proteins VPg and ProPol was associated with activation of the COX/PGE_2_ pathway. We further demonstrate that the production of PGE_2_ provides a protective effect against the antiviral effector mechanism of nitric oxide. Our findings uncover a new mechanism by which PSaV manipulates the host cell to provide an environment suitable for efficient viral growth, which in turn can be a new target for treatment of sapovirus infection.

## INTRODUCTION

Diarrhea is the second greatest cause of mortality in children worldwide (1). Viruses within the genera Norovirus and Sapovirus in the family Caliciviridae are significant causes of gastroenteritis in humans and animals, with noroviruses alone causing ∼200,000 deaths per annum in children <5 years of age (2, 3). Despite their socioeconomic impact, the fastidious nature of viruses within these genera has significantly hindered our understanding of their life cycles and the development of vaccines and therapeutics (4, 5). Porcine sapovirus (PSaV) is the only cultivable member of the genus Sapovirus and replicates in the presence of porcine intestinal contents or bile acids (4, 5). Therefore, PSaV serves as a robust model for studies on the sapovirus life cycle and for the development of therapeutic interventions (6).

The coexistence of viruses and their hosts imposes evolutionary pressure on both the virus and the host immune system. Therefore, viruses have evolved diverse strategies to create a suitable environment conducive to their existence by either activating or suppressing cellular pathways to facilitate replication. The cyclooxygenase-2 (COX-2)/prostaglandin E_2_ (PGE_2_) pathway is one of several host pathways that participate in the modulation of the host response to the infection and the replicative life cycle of viruses (7). For example, the activation of the COX-2/PGE_2_ pathway results in increased replication of cytomegalovirus (8, 9), but PGE_2_ inhibits the replication of parainfluenza 3 virus and adenovirus (10, 11). COXs convert arachidonic acid released by phospholipase A2- and C-mediated hydrolysis of plasma membrane phospholipids following exposure to diverse physiological and pathological stimuli into prostaglandins (PGs), prostacyclins, and thromboxanes (12, 13). Three forms of COX have been identified to date, with COX-1 and COX-2 the most widely studied. COX-1 is constitutively expressed and is known to synthesize various PGs, including PGE_2_, that participate in a diverse range of normal physiological processes, such as cytoprotection of the gastric mucosa, regulation of renal blood flow, bone metabolism, nerve growth and development, wound healing, and platelet aggregation (12, 13). In contrast, COX-2 is rapidly induced by various stimuli, including viral infection, and catalyzes the synthesis of various PGs, including PGE_2_, that have varied activities, including proangiogenic or antiapoptotic properties (12, 13). Some of the biological effects of PGE_2_ on immunity and inflammation are exerted through binding to G-protein-coupled receptors on the plasma membrane called E prostanoid receptors (14). PGE_2_ is recognized as the major prostanoid produced in immune and nonimmune cells and acts as a potent regulator of cell-cell interaction, antigen presentation, cytokine production, differentiation, survival, apoptosis, and cell migration (14).

This study examines the potential role of the COX/PGE_2_ pathway in the regulation of the sapovirus life cycle. We demonstrate that the COX/PGE_2_ pathway is induced during PSaV replication and that this induction occurs following the expression of the viral VPg and protease-polymerase (ProPol) proteins. We further demonstrate that the production of PGE_2_ provides a protective effect against the antiviral effector mechanism of nitric oxide (NO), uncovering a new mechanism by which enteropathogenic viruses manipulate the host cell to provide an environment suitable for efficient viral growth.

## RESULTS

### PSaV infection induces COX expression and leads to the production of PGE_2_.

**FIG 1:**
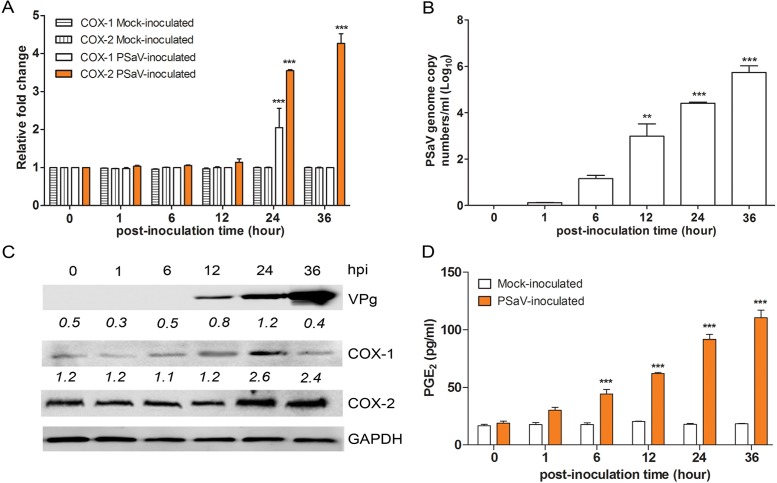
Induction of COX-1 and COX-2 by PSaV infection. (A and B) The expression of COX-1, COX-2, and PSaV viral RNA in LLC-PK cells infected with PSaV (MOI = 1 FFU/cell) was quantified by real-time PCR (qPCR). In the cases of COX-1 and COX-2, expression levels were normalized to β-actin and are depicted as the fold induction compared with that of the mock-inoculated cells. (C) The levels of the VPg, COX-1, COX-2, and GAPDH proteins were analyzed by Western blotting. GAPDH was used as a loading control. (D) The levels of PGE_2_ in the supernatants harvested at 36 hpi from PSaV-infected LLC-PK cells were determined by ELISA. The levels of PGE_2_ in the supernatants were compared between mock- and virus-inoculated groups. The data are presented as means and standard errors of the mean from the results of three independent experiments. Differences were evaluated using one-way analysis of variance (ANOVA). **, *P* < 0.001; ***, *P* < 0.0001.

To determine whether the COX/PGE_2_ pathway is activated during PSaV replication, we examined the impact of PSaV replication on COX gene expression. COX-2 mRNA and protein levels were markedly elevated at 24 and 36 h postinfection (hpi), concomitant with the increase in PSaV viral RNA and protein levels, whereas COX-1 levels were transiently increased at 24 hpi only (Fig. 1A to C). The level of PGE_2_ in the infected cell culture supernatant was also significantly elevated from 12 hpi (Fig. 1D).

**FIG 2:**
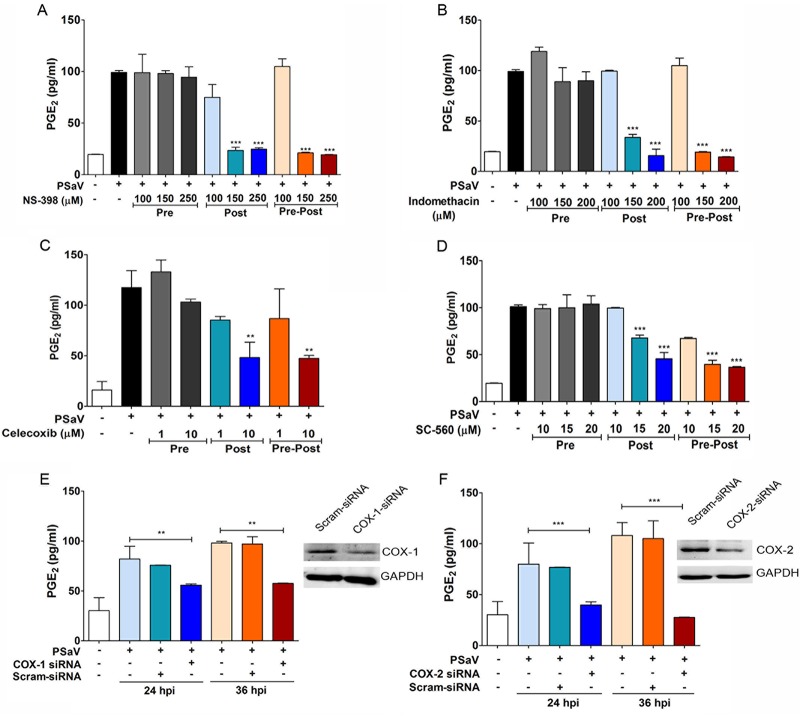
Effects of COX-2 inhibitors on PGE_2_ production during PSaV infection. (A to D) LLC-PK cells were treated with selective COX-2 inhibitors (NS398 and celecoxib), a nonselective COX inhibitor (indomethacin), and a selective COX-1 inhibitor (SC-560) as indicated prior to the addition of the virus inoculum (MOI = 1 FFU/cell), and then the inhibitor(s) was removed (Pre); after the addition of the virus inoculum, and then the inhibitor(s) was left for the duration of the infection (Post); or prior to the addition of the inoculum, as well as for the duration of the infection (Pre-Post). The levels of PGE_2_ in the supernatants harvested at 36 hpi were determined by ELISA. The levels of PGE_2_ in the supernatants of virus-infected cultures were compared between the mock- and chemical-treated groups. (E and F) Confluent LLC-PK cells were transfected with siRNAs against COX-1, COX-2, or scrambled siRNA (Scram-siRNA) prior to infection with PSaV (MOI = 1 FFU/cell). The supernatants were collected, and ELISA was conducted to determine the PGE_2_ concentrations. The levels of PGE_2_ in the supernatants were compared between mock- and siRNA-transfected groups. (Insets) Western blot analysis for COX-1, COX-2, and GAPDH was conducted with LLC-PK cells transfected with COX-1, COX-2, or scrambled siRNA. The data are presented as means and standard errors of the mean from the results of three independent experiments. Differences were evaluated using one-way ANOVA. **, *P* < 0.001; ***, *P* < 0.0001.

To confirm whether the observed increase in soluble PGE_2_ was a direct result of the PSaV-mediated induction of COX-1 and COX-2, the effect of selective or nonselective COX inhibitors on PGE_2_ production was examined (Fig. 2). Importantly, all the studies were performed at doses of inhibitors shown not to affect cell viability under the experimental conditions used (data not shown). The nonselective COX-1/2 inhibitor indomethacin and the selective COX-2 inhibitor NS-398 both inhibited PSaV-mediated PGE_2_ production in a dose-dependent manner when added either immediately after the removal of the virus inoculum (posttreatment) or during the entire course of the infection (pre/posttreatment) (Fig. 2A and B). In contrast, due to the reversible nature of the inhibitors, the pretreatment of cells and subsequent removal of the inhibitor prior to the addition of PSaV had no effect on the levels of PGE_2_ production (Fig. 2A and B). Similar results were also obtained with a range of other COX inhibitors (Fig. 2C and D and data not shown).

To further confirm direct roles for COX-1 and COX-2 in the production of PGE_2_ during PSaV infection, the effects of COX-1- or COX-2-specific small interfering RNAs (siRNAs) were also examined. Transfection of siRNA against COX-1 or COX-2 into LLC-PK cells reduced the expression levels of their respective target proteins. Although the inhibition of both intracellular proteins by siRNA transfection was not complete, the levels of PGE_2_ released from COX-1 and COX-2 siRNA-transfected cells were significantly reduced (Fig. 2E and F). These results confirmed that the induction of both COX enzymes was responsible for the observed increase in PGE_2_ from PSaV-infected cells. In addition, the level of siRNA-mediated reduction of COX-1 and COX-2 was sufficient to significantly reduce the production of PGE_2_.

### Inhibition of both COX enzymes negatively regulates PSaV replication.

**FIG 3:**
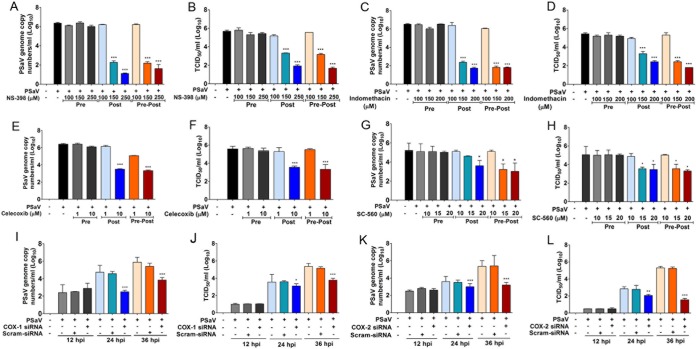
Inhibition of COXs attenuates PSaV replication. (A to H) LLC-PK cells were pretreated (Pre), posttreated (Post), or pre/posttreated (Pre-Post) with noncytotoxic doses of NS-398, indomethacin, celecoxib, and SC560. At 36 hpi with PSaV (MOI = 1 FFU/cell), cells were harvested, and the levels of viral RNA (A, C, E, and G) and the titer (B, D, F, and H) were determined by quantitative real-time PCR and TCID_50_, respectively. (I to L) LLC-PK cells were transfected with siRNAs against COX-1, COX-2, or scrambled siRNA before inoculation with PSaV (MOI = 1 FFU/cell). Samples were harvested at 36 hpi, and the levels of viral RNA (I and K) and the titer (J and L) were determined by quantitative real-time PCR and TCID_50_, respectively. The data are displayed as means and standard errors of the mean from the results of three independent experiments. Differences were evaluated using one-way ANOVA. *, *P* < 0.05; **, *P* < 0.001; ***, *P* < 0.0001.

**FIG 4:**
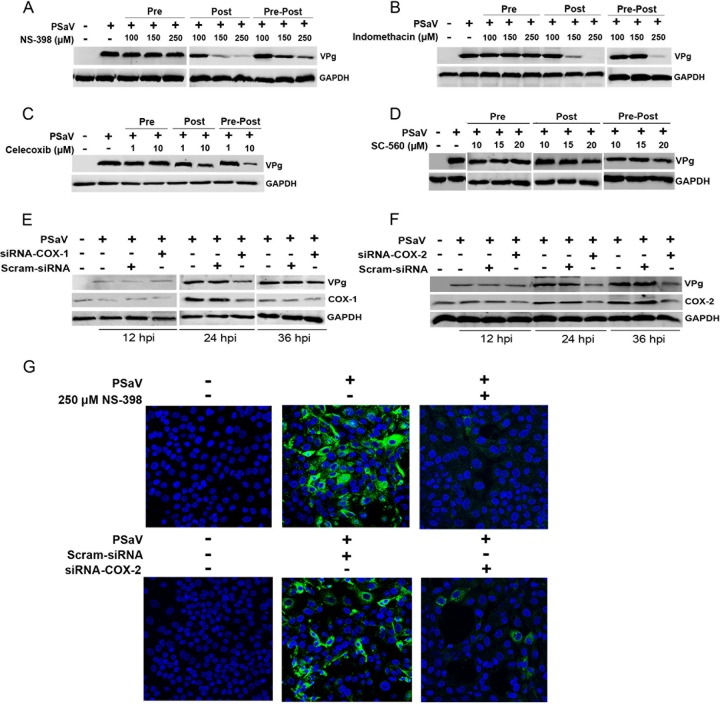
Effects of COX inhibitors or siRNA on PSaV replication. (A to D) LLC-PK cells were pretreated (Pre), posttreated (Post), or pre/posttreated (Pre-Post) with noncytotoxic doses of NS-398, indomethacin, celecoxib, and SC560. At 36 hpi with PSaV (MOI = 1 FFU/cell), cells were harvested, and the levels of viral VPg were determined by Western blot analyses. (E and F) LLC-PK cells were transfected with siRNAs against COX-1, COX-2, or scrambled siRNA before inoculation with PSaV (MOI = 1 FFU/cell). Samples were harvested and processed as described above. GAPDH was used as a loading control. (G) LLC-PK cells were infected with PSaV (MOI = 1 FFU/cell), and the effect of the COX-2 inhibitor NS-398 on viral antigen production was determined by confocal microscopy.

To determine the impact of COX induction on PSaV replication, we examined the effects of COX inhibitors and siRNAs on PSaV replication. Cells were either treated with inhibitors (selective or nonselective) or transfected with siRNAs specific for COX-1 or COX-2, and the effect on virus replication was monitored at 36 hpi by examining viral titers, as well as viral RNA and protein levels. There was no significant effect of COX inhibitors on PSaV replication when cells were pretreated but the inhibitors were removed prior to infection (pretreatment) (Fig. 3A to H and 4A to D). However, the inclusion of COX inhibitors following the removal of the virus inoculum (posttreatment) or during the entire course of infection (pre/posttreatment) resulted in a significant reduction in PSaV replication (Fig. 3A to H and 4A to D). The COX-1 inhibitor SC-560 reduced the levels of PSaV RNA and infectious virus by up to ∼10-fold at 36 hpi (Fig. 3G to H). In contrast, the COX-2-specific inhibitors NS-398 and celecoxib and the nonselective COX-1/2 inhibitor indomethacin showed more significant effects, typically leading to an ∼1,000-fold decrease in virus yield and RNA synthesis (Fig. 3A to F). Other COX-2-specific inhibitors (SC-58125, SC-236, and nimesulide) were also tested; however, less robust inhibition of PSaV replication was observed (data not shown).

The effects of COX-specific siRNAs on PSaV replication were also examined (Fig. 3I to L and 4E and F). Transfection of COX-2-specific siRNA had a more substantial effect on PSaV replication than that of COX-1 siRNAs, causing an ∼1,000-fold reduction in the viral titer with a concomitant decrease in viral RNA levels and viral protein production (Fig. 4E and F). Infection assays also demonstrated that either treatment of cells with NS-398 or transfection with COX-2-specific siRNAs resulted in a significant decrease in the number PSaV antigen-positive cells (Fig. 4G). Combined, these data suggest that both COX-1 and COX-2 enhance PSaV replication, possibly via the increased production of PGE_2_.

### Supplementation of PGE_2_ relieves COX inhibition of PSaV replication.

**FIG 5:**
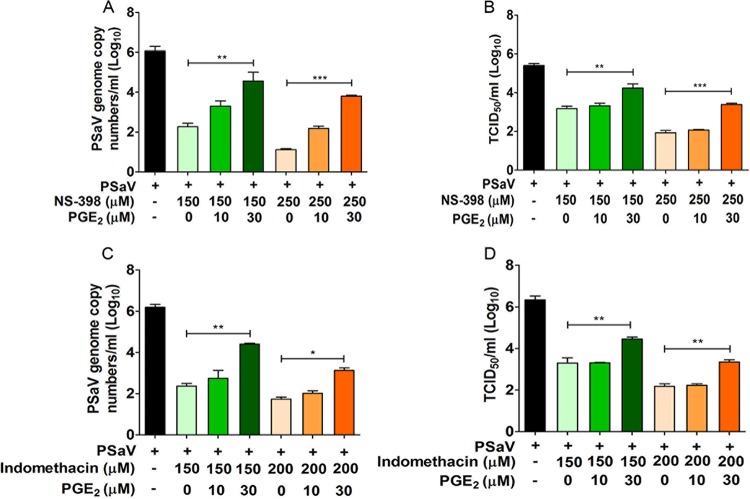
Addition of exogenous PGE_2_ reverses the effects of COX inhibitors on PSaV replication. LLC-PK cells were infected with PSaV (MOI = 1 FFU/cell), treated with noncytotoxic doses of NS-398 or indomethacin, and then supplemented with exogenous PGE_2_ in the maintenance medium. After 36 h postinfection, cells were harvested, and the levels of viral RNA synthesis (A and C) and the titer (B and D) were determined by quantitative real-time PCR and TCID_50_, respectively. The data are represented as means and standard errors of the mean from the results of three independent experiments. Differences were evaluated using one-way ANOVA. *, *P* < 0.05; **, *P* < 0.001; ***, *P* < 0.001.

If the proviral effect of COX gene induction on PSaV replication was due solely to the production of increased PGE_2_, then the addition of PGE_2_ would be expected to reverse the inhibitory effect of COX inhibitors on virus replication. To examine this possibility, the ability of exogenous PGE_2_ to restore PSaV replication after treatment with the nonselective COX-1/2 inhibitor indomethacin and the COX-2-specific inhibitor NS-398 was examined. The addition of exogenous PGE_2_ led to a dose-dependent restoration of both PSaV infectivity and viral RNA levels in cells treated with either inhibitor (Fig. 5). These results confirmed that PGE_2_, the final product of both COX enzymes, mediates the proviral effects of COX gene induction on PSaV replication.

### Bile acid does not influence COX-2 expression during PSaV infection.

**FIG 6:**
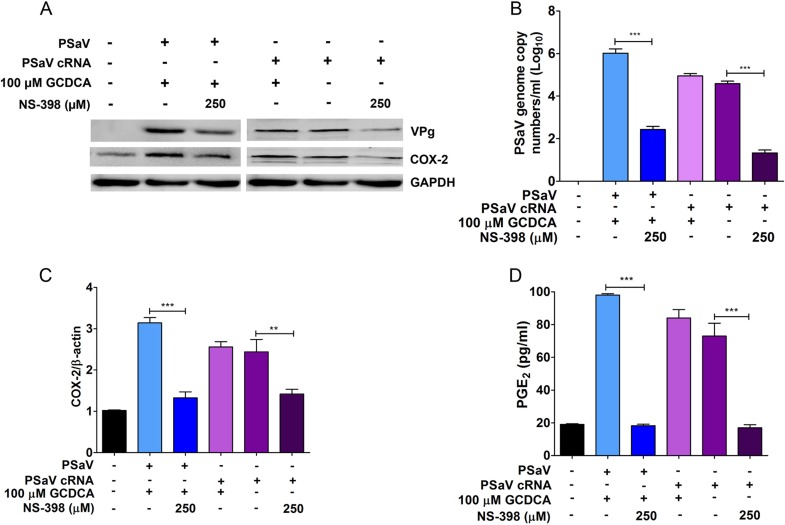
Bile acid GCDCA does not influence the expression of COX-2 during PSaV infection. (A to C) LLC-PK cells were either infected with PSaV (MOI = 1 FFU/cell) or transfected with 1 μg of *in vitro*-transcribed PSaV-capped RNA, and the effect of the COX-2 inhibitor NS-398 or the bile acid GCDCA was examined. Infected cells were harvested at 36 h postinfection, whereas transfected cells were harvested after 6 days posttransfection and subjected to Western blot analysis to assess viral protein production (A), as well as to qPCR for viral RNA (B) and COX-2 (C). (D) Supernatants were also collected for ELISA to quantify the levels of PGE_2_. The data are represented as means and standard errors of the mean from the results of three independent experiments. Differences were evaluated using one-way ANOVA. **, *P* < 0.001; ***, *P* < 0.001.

PSaV replication in cell culture relies on the presence of bile acids, including glycochenodeoxycholic acid (GCDCA), in the cell culture medium through a function that had previously been linked to an effect of bile acids on the innate immune response to infection (15). However, recent studies have indicated that this initial conclusion was incorrect, as PSaV remains sensitive to the type I interferon (IFN) response in the presence of bile acids (6) and bile acids function to promote virus uncoating (16). To determine whether bile acids have an effect on the induction of the COX-2/PGE_2_ pathway during PSaV replication, the induction of COX-2 and PGE_2_ was examined in the presence or absence of GCDCA following the transfection of the *in vitro*-transcribed and capped PSaV genome (Fig. 6). Transfected RNA was used to bypass any role of GCDCA during viral entry and uncoating (16). We observed that COX-2 and PGE_2_ were induced in cells transfected with *in vitro*-transcribed and capped PSaV genomic RNA, irrespective of whether GCDCA was present or absent (Fig. 6A). As expected, the COX-2 inhibitor NS-398 reduced expression of COX-2 and PGE_2_ and replication of PSaV (Fig. 6A to D). These data indicate that induction of COX-2 and PGE_2_ was a direct result of PSaV replication and was not due to any supplementary effect of GCDCA.

### PSaV VPg and ProPol activate the COX-2/PGE_2_ pathway.

**FIG 7:**
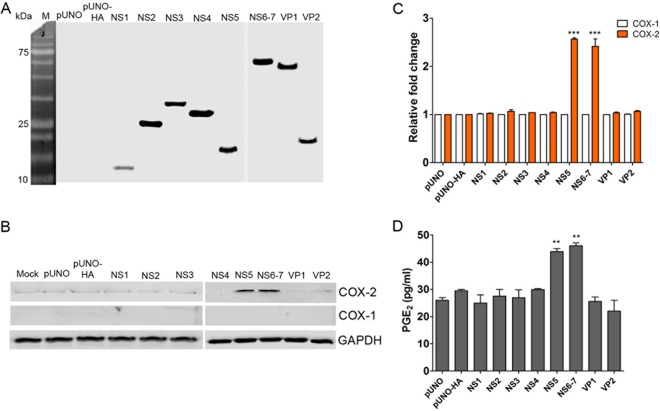
Role of PSaV proteins in stimulating COX-2 expression. (A and B) LLC-PK cells were transfected with 1 μg of pUNO plasmids containing each PSaV gene tagged with an HA epitope, as indicated in Materials and Methods. As controls, pUNO empty or HA-carrying (pUNO-HA) plasmids were transfected. At 36 h posttransfection (hpt), cells were harvested, and the expression levels of viral proteins (A), as well as COX-1 and COX-2 proteins (B), were determined by Western blotting. GAPDH was used as a loading control. (C) In the cases of COX-1 and COX-2, the expression levels were also quantified by real-time PCR and normalized to β-actin and are depicted as the fold induction compared with that of vehicle-transfected cells. (D) To determine the PGE_2_ concentration, supernatants were collected and ELISA was conducted. The levels of PGE_2_ in the supernatants were compared between vehicle- and PSaV gene-transfected groups. The data are presented as means and standard errors of the mean from three independent experiments. Differences were evaluated using one-way ANOVA. **, *P* < 0.001; ***, *P* < 0.0001.

Given our observation that PSaV replication was required for the induction of the COX-2/PGE_2_ pathway, we investigated whether the expression of viral protein alone was sufficient. LLC-PK cells were transfected with plasmids carrying each PSaV gene, including the NS1, NS2, NS3, NS4, NS5, NS6-7, VP1, and VP2 genes (17). Expression of each viral protein was confirmed via the detection of a hemagglutinin (HA) tag fused to the N terminus of each protein (Fig. 7A). Western blotting and quantitative-PCR (qPCR) analysis of the levels of COX-1 and COX-2 demonstrated that VPg or ProPol expression significantly enhanced the expression of COX-2 and led to an increase in PGE_2_ production (Fig. 7B to D).

### PGE_2_ blocks the antiviral effect of NO.

**FIG 8:**
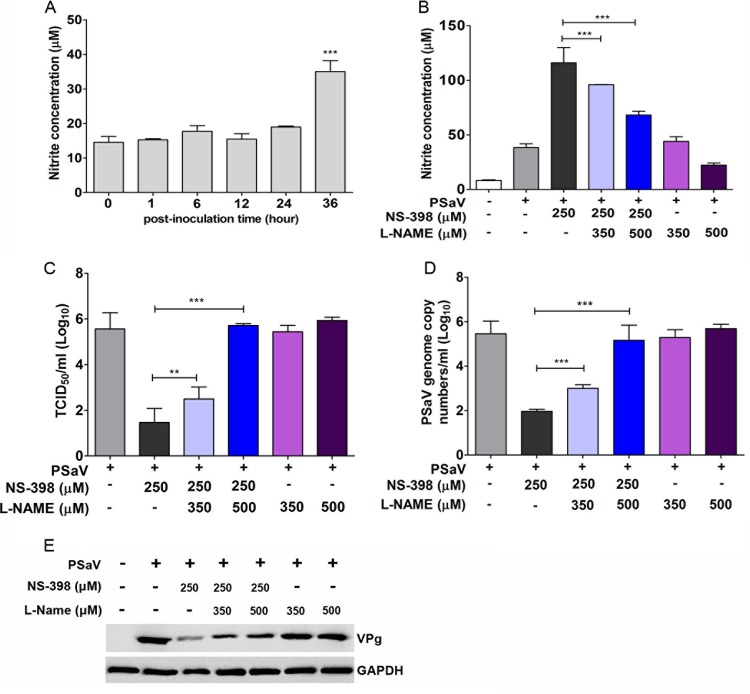
PGE_2_ blocks the antiviral effect of nitric oxide on PSaV infection. (A) Supernatants from mock- or PSaV-infected samples were collected, and the nitrite concentration was determined using the Griess reagent system, as described in Materials and Methods. LLC-PK cells were infected with PSaV (MOI = 1 FFU/cell) and subsequently treated with the COX-2 inhibitor NS-398 or the nitric oxide synthase inhibitor L-NAME, either singly or in combination. (B) The effect of inhibitor treatment on nitric oxide production was then determined as described for panel A. (C to E) The levels of viral titer (C), RNA (D), and protein (E) were determined by TCID_50_, real-time reverse transcription (RT)-PCR, and Western blot analyses, respectively. GAPDH served as the loading control. The data are means and standard errors of the mean from the results of three different independent experiments. Differences were evaluated by one-way ANOVA. **, *P* < 0.001; ***, *P* < 0.0001.

Previous studies have indicated that at least one of the effects of prostaglandin production is the regulation of NO production (18). NO is a key molecule involved in the host defense mechanism against various pathogens, including protozoans, parasites, fungi, bacteria, and viruses (19). NO also has a regulatory role at many stages of the development of inflammation (20). To determine if the PGE_2_ produced during PSaV replication impacted NO production, we first examined the level of NO produced during PSaV replication. PSaV-infected cells maintained low levels of NO prior to 36 hpi, when a significant increase in PGE_2_ production was observed (Fig. 8A). These data suggested that COX induction and the associated increase in PGE_2_ production may play important roles in the inhibition of NO production during PSaV replication.

To examine this possibility further, the effect of the COX-2 inhibitor NS-398 on NO production during PSaV infection was also examined (Fig. 8B). Inhibition of COX-2 activity was found to lead to a concomitant increase in NO production during PSaV replication, which could be reversed in a dose-dependent manner by the addition of the nitric oxide synthase (NOS) inhibitor L-NAME (Fig. 8B). The reversal of the effect of COX-2 inhibition by L-NAME also resulted in the subsequent restoration of PSaV infectivity levels, as well as PSaV RNA and protein levels (Fig. 8C to E). Collectively, these data suggest that the proviral effects of PGE_2_ produced by both the COX-1 and COX-2 enzymes as a result of PSaV infection are mediated by the inhibition of the antiviral effect of NO.

## DISCUSSION

During viral infection, numerous host inflammatory responses are induced, leading to the production of cellular effectors and soluble factors, such as IFNs, PGE_2_, and NO (21, 22). As obligate intracellular parasites, viruses must therefore subvert and/or avoid the host response to infection in order to complete their life cycle. As a result, pathogens, including viruses, have evolved a wide variety of mechanisms that enable the control of cellular pathways, evade the host immune response, and hijack signaling pathways to facilitate viral replication and pathogenesis. Here, we investigated the potential role of the COX/PGE_2_ pathway in the PSaV life cycle and the control of infection. We found that the COX/PGE_2_ pathway was activated during the PSaV life cycle and that this activation had proviral effects via the inhibition of NO production. Furthermore, we determined that expression of the VPg and ProPol proteins of PSaV was sufficient to induce COX expression and PGE_2_ production.

Among the soluble factors produced during the response of host cells to viral infection, NO is well known in the antiviral repertoire (23–26). In the immunological system, inducible NOS (iNOS) is induced by cytokines at the transcriptional level primarily in macrophages, neutrophils, epithelial cells, and hepatocytes, where NO is produced at high concentrations (27, 28). iNOS, the enzyme responsible for the production of NO, can be regulated by the COX/PGE_2_ pathway (25). NO production is known to restrict vesicular stomatitis virus (VSV) infection (23), an effect that is thought to be offset by the induction of PGE_2_ production during VSV replication *in vivo* (18). Here, we showed that activation of the COX-2/PGE_2_ pathway also enhances PSaV replication through an inhibitory effect on NO production.

NO production is significantly enhanced in patients suffering from gastroenteritis (24), and this has been observed in norovirus and rotavirus infections in children (26). NO secretion can be triggered *in vitro* and *in vivo* by rotavirus nonstructural protein 4 (NSP4) (28–30), which in turn may cause diarrhea by elevating intestinal permeability (31, 32) and regulating intestinal motility (33) and intestinal ion transport (34, 35). In contrast to rotavirus infection (28–30), little NO production was observed during PSaV replication in LLC-PK cells, possibly due to the synthesis of PGE_2_, suggesting that NO may not be involved in sapovirus-induced diarrhea. However, it is important to note that we cannot fully rule out a potential role for NO in PSaV pathogenesis *in vivo*, where the cellular response to infection is influenced by numerous cell types and may not be entirely reproduced in immortalized cells *in vitro*.

In a few instances, viral proteins have been shown to stimulate activation of the COX/PGE_2_ pathway, often by acting either directly or indirectly as a transcriptional transactivator of COX-2 gene expression (36–39). Among the viral proteins for which this activity has been reported, the hepatitis C virus (HCV) protein NS3, a viral serine protease, is known to enhance the COX-2/PGE_2_ pathway by activating multiple signaling pathways (40). In addition, the severe acute respiratory syndrome coronavirus (SARS-CoV) nucleocapsid protein also activates the expression of the COX-2/PGE_2_ pathway by binding directly to regulatory elements for NF-κB and CCAAT/enhancer binding protein (39). In the present study, we demonstrated that expression of the PSaV VPg or ProPol protein in isolation was sufficient to lead to activation of the COX-2/PGE_2_ pathway. The mechanism behind this activation remains to be determined and is the subject of future study.

In the present study, the levels of COX-1/2 mRNA and proteins were increased in response to PSaV infection, so we evaluated the effects of COX-1-specific, COX-2-specific, and nonselective COX-1/2 inhibitors on PSaV replication. Among the inhibitors tested, the COX-2-specific inhibitors NS-398 and celecoxib and the nonselective COX-1/2 inhibitor indomethacin exerted stronger anti-PSaV effects than other inhibitors, with ∼1,000-fold decreases in virus yield and RNA synthesis. However, other COX-2-specific inhibitors had weaker inhibitory effects on PSaV replication, most likely due to their less potent activity and the fact that higher concentrations required for effective COX inhibition resulted in cell toxicity observed during their use. In addition, more robust inhibitory effects of the COX-2-specific inhibitor NS-398 and the COX-1/2 nonselective inhibitor indomethacin on PSaV replication was observed in posttreatment than in pre/posttreatment. The reason why posttreatment with these inhibitors has a stronger effect than pre/posttreatment remains unknown. One possibility is that it could be an off-target effect of using high doses of the inhibitors in LLC-PK cells, although there are no published reports of these kinds of phenomena.

In the present study, we observed only a transient increase in COX-1 levels during PSaV infection, whereas COX-2 induction was more significantly induced and the induction was sustained during the later stage of the viral life cycle. However, the dramatic effects of the nonselective COX inhibitor indomethacin, the COX-2-specific inhibitor NS-398, and the COX-specific siRNAs support the hypothesis that both the COX-1 and COX-2 enzymes play roles in PSaV replication.

In conclusion, our results demonstrate a crucial role of the COX/PGE_2_ pathway in the regulation of NO production during PSaV replication, which provides an environment suitable for efficient PSaV growth. In addition, our data indicate that pharmacological targeting of COX-2 could provide a potential targeting strategy for the control of sapovirus infection, facilitating the antiviral effect of NO production. Further studies are required to determine if targeting the COX/PGE_2_ pathway *in vivo* has a negative impact on PSaV pathogenesis.

## MATERIALS AND METHODS

### Cells and virus.

LLC-PK porcine kidney cells obtained from the American Type Culture Collection (ATCC) were maintained in Eagle's minimal essential medium (EMEM) containing 10% fetal bovine serum (FBS), 100 U/ml penicillin, and 100 μg/ml streptomycin. The tissue culture-adapted PSaV strain Cowden was recovered from the full-length infectious clone pCV4A and was propagated in LLC-PK cells supplemented with bile acid (15).

### Chemicals and antibodies.

Celecoxib, NS-398, SC-58125, SC-236, nimesulide, and SC-560 were purchased from Cayman Chemical (Ann Arbor, MI, USA). GCDCA, dimethyl sulfoxide (DMSO), L-NAME, MDL-12330A, and indomethacin were from Sigma-Aldrich (St. Louis, MO, USA). COX-1 siRNA, COX-2 siRNA, and scrambled siRNA were purchased from Santa Cruz Biotechnology, Inc. (Santa Cruz, CA, USA). Monoclonal antibody (MAb) against mouse COX-1 and polyclonal antibody against rabbit COX-2 were obtained from Abcam (Cambridge, MA, USA). Mouse MAb against HA tag was purchased from OriGene (Rockville, MD, USA). Synthetic PGE_2_ was purchased from Tocris Bioscience (Ellisville, MO, USA). The anti-PSaV capsid MAb and the anti-PSaV VPg polyclonal antibody were previously described (41). The secondary antibodies used were horseradish peroxidase-conjugated goat immunoglobulin against rabbit IgG (Cell Signaling, Beverly, MA, USA) and mouse IgG (Santa Cruz) and fluorescein isothiocyanate (FITC)-conjugated goat immunoglobulin against rabbit IgG (Jackson Immuno Research Laboratory, West Grove, PA, USA).

### Cytotoxicity assay.

The cytotoxicity of the chemicals used in this study was determined using the 3-(4,5-dimethylthiazol-2-yl)-2,5-diphenyl tetrazolium bromide (MTT) assay (42, 43) according to the manufacturer's instructions. Briefly, cells in 96-well plates were incubated with medium containing different concentrations of various chemicals for 24 h. After removal of the medium, 200 μl of MTT solution was added to each well and incubated for 4 h at 37°C in a CO_2_ incubator. Each well was supplemented with 150 μl of DMSO and incubated at room temperature for 10 min. The absorbance as an optical density (OD) was read in an enzyme-linked immunosorbent assay (ELISA) reader at 570 nm. The percent cell viability was calculated using the following formula: [(OD_sample_ − OD_blank_)/(OD_control_ − OD_blank_)] × 100. Nontoxic concentrations of each chemical were used in the study.

### Treatment of LLC-PK cells with inhibitors and chemicals.

LLC-PK cells were grown in 6- or 12-well plates to attain the desired confluence. The chemicals and inhibitors were dissolved in DMSO to make a 10 mM stock concentration. When appropriate, a series of dilutions were made by diluting the appropriate volumes of chemical or inhibitor stocks in EMEM. Treatment groups were typically as follows: mock treatment, pretreatment, posttreatment, and pre/posttreatment. Confluent LLC-PK cells were pretreated with various concentrations of the inhibitors for 24 h. The cells were washed with phosphate-buffered saline (PBS) (pH 7.4) and inoculated with PSaV at a multiplicity of infection (MOI) of 1 fluorescent focus unit (FFU)/cell. For posttreatment groups, different concentrations of inhibitors were added to the maintenance medium after the virus adsorption step. For the pre/posttreatment groups, LLC-PK cells were pretreated with different concentrations of inhibitors for 24 h. After the removal of the inhibitors, the cells were washed twice with PBS and inoculated with PSaV. The inhibitors were again added at the end of the virus adsorption period.

### Plasmid constructs and cell culture.

Each of the regions coding for the PSaV proteins NS1, NS2, NS3, NS4, NS5, NS6-7, VP1, and VP2 (17) was amplified from the full-length infectious clone pCV4A by PCR assays with primer pairs containing SalI and NheI restriction enzyme sites (Table 1). Each forward primer specific for the above-mentioned genes had N-terminal HA tag sequences (Table 1). Each amplicon was purified with PuriGel (Invitrogen, Waltham, MA, USA) following the manufacturer's instructions and subcloned into the pUNO cloning vector (Invivogen, San Diego, CA, USA). All the amplified regions were verified by Sanger sequencing. LLC-PK cells grown in 6-well plates were individually transfected with plasmid constructs with different viral genes inserted or empty vectors using the Lipofectamine 2000 reagent (Invitrogen) following the manufacturer's instructions. Cells were harvested at different posttransfection points and subjected to quantitative real-time PCR and Western blot analysis using the anti-HA antibody.

**TABLE 1:** Oligonucleotide primers used in the study

Target gene	Primer name	Orientation^*a*^	Sequence (5′–3′)^*b*^	Region (nt)^*e*^	Size (bp)
p11	pUNO-HA-p11	F	gtg gtcgac **atg** *TAC CCA TAC GAT GTT CCA GAT TAC GCT* GCT AAT TGC CGT CCG TTG CCT ATT GGG	13–39	165
		R	atc gctagc TCA TTG CGC CAC AAA CAC GTC	177–160	
p28	pUNO-HA-p28	F	ttt gtcgac **atg** *TAC CCA TAC GAT GTT CCA GAT TAC GCT* GGG GTG GTG GAT GAT TTC TTC CGC CCC	178–204	762
		R	atc gctagc TCA CTG CGG CGT GTA GAG GC	939–923	
p35	pUNO-HA-p35	F	tac gtcgac **atg** *TAC CCA TAC GAT GTT CCA GAT TAC GCT* GCA GGC AAT GAT CTC ATC ATA TTG GGG	940–966	1,017
		R	gac gctagc TCA CTC GCT GTT GTA CTT C	1956–1941	
p32	pUNO-HA-p32	F	tac gtcgac **atg** *TAC CCA TAC GAT GTT CCA GAT TAC GCT* GCC GCT GAT GTC AAA CAT CTA TGG TTC	1957–1983	855
		R	ttt gctagc TCA CTC GCT AAG CGT GTT TTC	2811–2794	
VPg	pUNO-HA-VPg	F	ac gtcgac **atg** *TAC CCA TAC GAT GTT CCA GAT TAC GCT* GCG AAA GGG AAA AAC AAA CGC GGA CGT	2812–2838	338
		R	gctagc TCA CTC ACT GTC ATA GGT GTC ACC TTT	3150–3133	
ProPol	pUNO-HA-ProPol	F	gtcgac **atg** *TAC CCA TAC GAT GTT CCA GAT TAC GCT* GGG CGT GGA TAC GTG GTA CCC ATG AC	3151–3177	1,995
		R	gctagc TCACTCCATCACGAACACTTCTGGCTCTTC	5145–5128	
VP1	pUNO-HA-VP1	F	gtcgac **atg** *TAC CCA TAC GAT GTT CCA GAT TAC GCT* GAG GCG CCT GCC CCA ACC CGT TCG GTT	5144–5169	1,627
		R	gctagc TCATCGTGAGCTGTGAATGGACCTTCC	6771–6753	
VP2	pUNO-HA-VP2	F	ctc gtcgac **atg** *TAC CCA TAC GAT GTT CCA GAT TAC GCT* AGT TGG ATT GCA GGA GCA ATG CAG GGC	6774–6800	492
		R	agg gctagc TCA TCA CAC TTT GCT GTG AGT G	7265–7247	
VPg		F	CAA ACG CGG ACG TGG TGC TCG	2826–2846	145
		R	TGA TGC GCC TGA CAG TGC GCG	2970–2950	
COX-1^*c*^		F	CCG GAG GAA GTT CAT ACC TGA CCC	253–275	108
		R	GCC AGG ACC CAT CTT GCC AGA	360–340	
COX-2^*d*^		F	CAC CCA TGG GTG TGA AAG GGA GG	181–203	201
		R	CCA AAG GAC AGG GCC ATG GGG	381–361	

a F, forward; R, reverse.

b Lowercase underlined letters, restriction enzyme sites (forward primers, SalI; reverse primers, NheI); italic letters, polypeptide hemagglutinin sequences; lowercase boldface letters, start codons. The lowercase letters at the start of the forward primers of the PSaV genome are overhang sequences.

c Derived from partial sequence of porcine COX-1 (GenBank accession no. AF207823.1).

d Derived from partial sequence of porcine COX-2 (GenBank accession no. AF207824.1).

e nt, nucleotides.

### *In vitro* transcription and RNA transfection.

LLC-PK cells were seeded in 6-well or 24-well plates and transfected with 1 μg of capped *in vitro*-transcribed PSaV RNA using Lipofectamine 2000 (Invitrogen). The capped *in vitro* transcripts were derived from the full-length PSaV cDNA clone pCV4A (44) with the mMessage mMachine kit (Ambion, Austin, TX, USA) following the manufacturer's instructions. Transfections were performed for 4 h, and the medium was replenished with EMEM supplemented or not with 200 μM GCDCA. Six days posttransfection, the cells were lysed, harvested, and subjected to immunoblotting and qPCR to analyze COX-1, COX-2, and PSaV VPg levels.

### siRNA transfection.

LLC-PK cells were cultured in 6- or 12-well culture plates at 70 to 80% confluence and transfected with siRNA (80 pmol of COX-1, COX-2, and scrambled control siRNA) using the Lipofectamine 2000 reagent (Invitrogen) following the manufacturer's instructions. The cells were then infected with PSaV at an MOI of 1 FFU/cell. After 1 h, unadsorbed viruses were removed and the cells were maintained in EMEM with 2.5% FBS and 100 μM GCDCA. Cells were harvested at different time points and subjected to qPCR and median (50%) tissue culture infective dose (TCID_50_) and Western blot analyses.

### Preparation of cell extracts and Western blot analysis.

Confluent LLC-PK cells in 6-well plates infected or not with PSaV, treated or not with chemicals or inhibitors, transfected or not with siRNAs, or transfected or not with each gene construct of PSaV were harvested at different time points. The cells were washed twice with PBS and lysed with a cell extraction buffer (Invitrogen) supplemented with protease and phosphatase inhibitors (Roche, Basel, Switzerland). Total cell lysates were denatured and resolved in sodium dodecyl sulfate (SDS)-polyacrylamide gels. The resolved proteins were transferred to nitrocellulose blotting membranes (Amersham Protran; GE Healthcare Life Science, Germany) and immunoblotted with primary antibodies specific for COX-1, COX-2, glyceraldehyde 3-phosphate dehydrogenase (GAPDH), HA, or PSaV VPg. Secondary antibodies against rabbit or mouse IgG were applied after the primary antibody. Immunoreactive bands were developed using an enhanced chemiluminescence reaction kit (DoGen, Seoul, South Korea), and images were taken using the Davinch-Western imaging system (Young Ltd., Kang-Nam, Seoul, South Korea). To confirm equal protein loading, the blotting membranes were also incubated with an antibody against GAPDH, and its reactivity was compared with the intensities of target bands. The quantification of the protein densities for COX-1 and COX-2 was performed using Image Studio Lite (LI-COR Biotechnology, Lincoln, NE, USA) and was normalized to the corresponding density of GAPDH in the same samples.

### RNA isolation.

To quantify intracellular RNA levels of signaling molecules, mock- or PSaV-infected, chemical- or inhibitor-treated, or siRNA-transfected cells were washed twice with PBS, scraped, and collected in clean microtubes. Samples were centrifuged at 10,000 rpm for 10 min, and total RNA was isolated using the PureLink RNA minikit (Ambion Life Technologies, Carlsbad, CA, USA) following the manufacturer's instructions. To quantify PSaV RNA, mock- or PSaV-infected, chemical- or inhibitor-treated, or siRNA-transfected cells were freeze-thawed three times, and the cell debris was spun down at 2,469 × *g* for 10 min at 4°C. The supernatants, along with the remaining bulk samples, were collected and stored at −80°C until they were used. Total RNA was extracted from supernatants using an RNeasy kit (Qiagen) following the manufacturer's instructions. The RNA concentrations were spectrophotometrically determined at 260 nm using a BioPhotometer plus (Eppendorf, Hamburg, Germany).

### Quantitative real-time PCR.

cDNAs were prepared by using 1 μg of RNA and reverse transcribed using random hexamers (Promega, Madison, WI, USA). The oligonucleotide primers used in the quantitative real-time PCR were designed from the published sequences of COX-1, COX-2, and PSaV VPg (Table 1). Reaction mixtures were set up in 25-μl volumes containing 10 pmol of forward and reverse primers, cDNA, and Topreal qPCR 2× premix (Enzynomics, Daejon, South Korea). For COX-1 and COX-2, the amplification profile was as follows: 1 cycle of initial denaturation at 95°C for 10 min and 45 cycles of denaturation at 95°C for 10 s, primer annealing at 55°C for 30 s, and extension at 72°C for 45 s. The amplification profile for VPg included denaturation at 95°C for 10 min, followed by 40 cycles of denaturation at 95°C for 10 s, primer annealing at 60°C for 20 s, and extension at 72°C for 20 s. Relative COX-1 and COX-2 expression levels were calculated using 2^−ΔΔ^^*CT*^ (45). Samples were normalized to the quantity of β-actin genes. The copy number of the VPg gene was calculated using 10-fold dilutions of a known amount of pCV4A to generate the standard curve.

### TCID_50_ assay.

The TCID_50_ assay was performed as previously described (6). Briefly, 10-fold serial dilutions of clarified virus supernatants were prepared in EMEM. Of these dilutions, 200 μl was inoculated into monolayers of LLC-PK cells grown on 96-well plates supplemented with 200 μM GCDCA and incubated at 37°C in a 5% CO_2_ incubator. Virus titers were calculated at 6 days postinfection and expressed as TCID_50_/ml values by the method of Reed and Muench (46).

### Determination of infectivity titers by immunofluorescence assay.

Infectivity assays were carried out as described previously (41). Briefly, confluent monolayers of cells on a confocal dish were treated with various inhibitors or chemicals as described above. Mock-treated or inhibitor-treated cells were infected with PSaV at an MOI of 1 FFU/cell and incubated at 37°C for 1 h. The cells were washed three times with PBS, moved to maintenance medium, and then incubated for 36 h at 37°C prior to being fixed with 4% formaldehyde in PBS.

Immunofluorescence assays were performed as previously reported (41). Briefly, fixed cells in 8-well chamber slides were permeabilized by the addition of 0.2% Triton X-100, incubated at room temperature for 10 min, and washed with PBS containing 0.1% newborn calf serum (PBS-NCS). The chamber slides were supplemented with anti-PSaV capsid (1:40 dilution) MAb and then incubated at 4°C overnight. The cells were then washed three times with PBS-NCS, and FITC-conjugated goat secondary antibody (diluted 1:100) was added. After washing with PBS, the chambers were mounted with SlowFade Gold antifade reagent (Life Technologies, Eugene, OR, USA) containing DAPI (4′,6-diamidino-2-phenylindole) solution for nuclear staining. Infected cells were observed with an LSM 510 confocal microscope and analyzed using LSM software (Carl Zeiss). To calculate the percentages of antigen-positive cells, 1,000 cells in each well were counted using a 40× objective and a 10× eyepiece, yielding a final magnification of ×400. The numbers of antigen-positive cells in mock- and drug-treated or scrambled RNA and siRNA against the COX-2 gene were compared.

### Determination of NO concentrations.

The concentrations of NO in culture supernatants of mock- or PSaV-infected cells in the presence or absence of chemicals or inhibitors were determined by assaying nitrite, one of its stable end products. The collected supernatants were centrifuged to remove cell debris. The assay was done using the Griess reagent system (Promega, Madison, WI, USA) according to the manufacturer's instructions. Briefly, equal volumes of each experimental sample and sulfanilamide solution were incubated at room temperature for 10 min. An equal volume of *N*-1-naphthylethylenediamine dihydrochloride solution was then added to all the wells and incubated for 10 min. The absorbance was read at 540 nm in a plate reader. The nitrite concentration of each sample was determined by comparing it with a generated nitrite standard and calculated by linear regression analysis.
